# Isolation and characterization of an enterovirus G strain causing diarrhea and gut microbiota dysbiosis in piglets

**DOI:** 10.3389/fcimb.2025.1699507

**Published:** 2025-11-17

**Authors:** Benqiang Li, Jie Tao, Jinghua Cheng, Ying Shi, Pan Tang, Qi Li, Lilei Lv, Huili Liu

**Affiliations:** 1 Institute of Animal Husbandry and Veterinary Medicine, Shanghai Academy of Agricultural Sciences, Shanghai, China; 2 Shanghai Key Laboratory of Agricultural Genetic Breeding, Shanghai, China; 3 Shanghai Engineering Research Center of Pig Breeding, Shanghai, China

**Keywords:** EV-G, genetic evolution analysis, recombination, pathogenicity, gut microbiota

## Abstract

**Background:**

Enterovirus G (EV-G) is a significant causative agent of diarrhea in piglets, complicating differential diagnosis and impeding effective control of porcine diarrheal diseases. Enhanced clinical surveillance and pathogenicity assessment of EVG are essential to inform disease prevention strategies.

**Methods:**

The EV-G strain was isolated and identified in MA-104 cells using plaque assay, immunofluorescence staining, viral replication kinetics analysis, and transmission electron microscopy. The complete genomic sequence was obtained for phylogenetic and recombination analysis. Five-day-old piglets were orally challenged with the prevalent EV-G strain, and pathogenicity was assessed based on diarrhea scoring, viral load quantification in feces and tissues, and histopathological examination. Gut microbiota dynamics were profiled via high-throughput 16S rRNA sequencing pre- and post-infection.

**Results:**

A recombinant G1 genotype EV-G strain, designated EVG-SH-2024, was successfully isolated. Phylogenetic analysis indicated its closest amino acid identity (94.7%) with KF985175, while recombination analysis suggested origin from genetic exchange between KF985175 and MN734577. Inoculation with EVG-SH-2024 induced mild diarrhea and persistent viral shedding in piglets, accompanied by significant tissue damage including neuronal vacuolation in the brain, pulmonary consolidation, and intestinal inflammatory infiltration. 16S rRNA sequencing revealed substantial gut microbiota dysbiosis post-infection, marked by an increase in pathogenic bacteria and a decline in beneficial microbes. Notably, the top ten differentially abundant species were predominantly short-chain fatty acid (SCFA)-producing bacteria, whose abundance showed a significant Spearman’s rank correlation with pathogenicity indicators.

**Conclusion:**

This study reports the first isolation and characterization of a G1 subtype EV-G strain in Shanghai, China. Integrated pathogenicity and microbiota analyses demonstrated that EV-G infection reduces the abundance of SCFA-producing bacteria, with this reduction significantly correlated with disease severity. These findings suggest a potential role of SCFAs in EV-G pathogenesis and provide a basis for developing microbiota-targeted interventions against EV-G infection.

## 1 Introduction

Porcine enterovirus (PEV) is a major viral pathogen that contributes to piglet diarrhea and represents a significant threat to swine herd health. Historically, PEV was classified into 13 serotypes (PEV-1 to -13) and grouped into three genera: Teschovirus, Sapelovirus, and Enterovirus (Krumbholz et al., 2002). PEV-1 to -7 and PEV-11 to -13 belong to the genus Teschovirus, PEV-8 to Sapelovirus, and PEV-9 and PEV-10 to Enterovirus G (EV-G) (Walker et al., 2021). EV-G is a member of the family *Picornaviridae*, order *Picornavirales*, comprising a highly diverse group of 20 recognized genotypes (EV-G1 to EV-G20) widely distributed in pig populations worldwide (Bhat et al., 2024).

EV-G infection in pigs is associated with diverse clinical manifestations, including neurological signs (polioencephalomyelitis), respiratory disease (pneumonia), enteric disease (diarrhea), and reproductive disorders (SMEDI syndrome: stillbirth, mummified fetus, embryonic death, and infertility). The virus was first isolated in the United Kingdom between 1972 and 1976 (Knowles et al., 1979) and has since been detected in fecal samples from both diarrheic and healthy pigs globally (Schyns et al., 2025). In China, EV-G detection remains limited, with reports from Heilongjiang (Ibrahim et al., 2023), Jiangsu (Wang et al., 2018), Yunnan (Zhu et al., 2024), Guangxi (Mi et al., 2021), and Sichuan (Xiao et al., 2023), restricting current understanding of its epidemiology and pathogenicity.

The gut microbiota plays a central role in host health, contributing to immune regulation, nutrient metabolism, and disease modulation in both humans and animals (Paul et al., 2025). Viral infections, such as those caused by coronaviruses (CoVs), can profoundly alter the composition, structure, and metabolic activity of the gut microbiota, which in turn influences viral pathogenesis and host antiviral immunity (Ishizaka et al., 2024; Zhu et al., 2025). Consequently, microbiota-targeted approaches are increasingly being explored as preventive and therapeutic strategies for CoV-associated disease (Ng et al., 2023). Porcine epidemic diarrhea virus (PEDV), transmissible gastroenteritis virus (TGEV), and porcine deltacoronavirus (PDCoV) induce intestinal inflammation, alter oxygen availability in the gut, and modulate microbial colonization and metabolism (Song et al., 2017; Zhang et al., 2024; Chen et al., 2025). Such infections can indirectly reshape the gut microbiota through immune-mediated mechanisms, aggravating intestinal dysfunction and disease progression.

In this study, we report the first isolation and characterization of a recombinant G1 subtype EV-G strain (EVG-SH-2024) from Shanghai, China. Pathogenicity assays in piglets demonstrated that EVG-SH-2024 caused mild diarrhea accompanied by marked intestinal inflammation. 16S rRNA gene sequencing revealed profound gut microbiota dysbiosis, with a significant reduction in short-chain fatty acid (SCFA) -producing bacterial populations, compromising intestinal barrier function and triggering inflammatory responses that culminated in diarrhea. Our findings not only establish a mechanistic link between EV-G pathogenesis and gut microbiome perturbations but also underscore the potential of microbiota–targeted interventions in controlling porcine diarrheal diseases.

## 2 Materials and methods

### 2.1 Virus isolation and proliferation

In December 2024, ten diarrheic fecal samples from piglets were collected from a pig farm in Shanghai. RT-PCR detection results showed a PoRV positivity rate of 60% and an EV-G positivity rate of 30%, with one sample identified as a co-infection of PoRV and EV-G. In this study, we selected one of the EV-G single-infection samples for viral identification. The EV-G–positive fecal sample was filtered to remove particulate matter and treated with penicillin–streptomycin solution (HyClone, Thermo Fisher Scientific, USA), followed by overnight incubation at 4°C. The treated sample was inoculated onto confluent MA-104 cell monolayers grown in 25 cm² flasks containing MEM medium supplemented with 0.5 µg/mL TPCK-treated trypsin (Sigma-Aldrich, St. Louis, MO, USA). After a 2h adsorption period at 37°C in 5% CO_2_, the inoculum was removed and replaced with fresh maintenance medium. The cultures were monitored daily for cytopathic effects (CPE). At 72h post-infection (hpi), cultures exhibiting significant CPE were harvested and subjected to at least five serial blind passages in fresh MA-104 monolayers under identical conditions (Mi et al., 2021).

### 2.2 Phaque purification assay

Plaque purification of the EV-G isolate was conducted as previously described (Ibrahim et al., 2022). Briefly, serial 10-fold dilutions of the viral suspension were inoculated onto confluent MA-104 cell monolayers in 6-well plates. After 2 h of adsorption at 37°C in 5% CO_2_, the inoculum was aspirated, and the cells were overlaid with 1% low–melting point agarose in MEM medium containing 0.5 µg/mL TPCK-treated trypsin. The plates were gently swirled for uniform distribution and allowed to solidify at room temperature. Following incubation at 37°C in 5% CO_2_ for 5–7 days, discrete plaques were visualized under a microscope, excised with sterile tips, and suspended in 1 mL MEM. Three successive rounds of plaque purification were performed by re-inoculating excised plaques onto fresh MA-104 monolayers. After each passage, viral clones were propagated in MEM containing 0.5 µg/mL TPCK-trypsin. Plaque formation was confirmed by fixing monolayers with 4% formaldehyde and staining with 0.5% crystal violet.

### 2.3 Immunofluorescent staining

MA-104 cells infected with EV-G for 48h were washed with PBS and fixed with 75% ethanol for 20min at –20°C. Cells were incubated with EV-G polyclonal antibody (1:500) at 37°C for 1h, followed by FITC-labeled sheep anti-mouse IgG secondary antibody (1:2000) (Invitrogen, Carlsbad, CA, USA) at 37°C for 1h. Nuclei were counterstained with ProLong™ Diamond Antifade Mountant (Invitrogen, Carlsbad, CA, USA), and images were acquired using a Zeiss fluorescence microscope.

### 2.4 Viral replication kinetics curve

To determine the growth kinetics of the purified EV-G clone, MA-104 cells were seeded in 24-well plates to ~90% confluence and infected at a multiplicity of infection (MOI) of 0.01. After 1 h of adsorption, unbound virus was removed, and the cells were maintained in fresh MEM supplemented with TPCK-trypsin. Supernatants were collected at 12, 24, 36, 48, 60, 72, and 84 hpi, and virus titers (50% tissue culture infective dose/0.1mL, TCID_50_/0.1mL) were measured following the Reed-Muench method (Ibrahim et al., 2023).

### 2.5 Transmission electron microscopy

MA-104 cells infected with EV-G were harvested when the CPE exceeded 80%. The culture supernatant was clarified and subjected to ultracentrifugation at 40, 000 × g for 4h at 4°C (Beckman Coulter Optima XE-100, USA). The pellets were resuspended in 1 mL TNE buffer (Solarbio, Beijing, China) and incubated overnight at 4°C. For transmission electron microscopy (TEM), 20 µL of viral suspension was placed on a carbon-coated 200-mesh copper grid and adsorbed for 5min, followed by negative staining with 2% phosphotungstic acid (PTA) for 2 min. Excess stain was removed, the grids were air-dried, and viral morphology was examined using a Hitachi HT7800 TEM (Hitachi High Technologies, Japan) at Wuhan Servicebio Technology Co., Ltd.

### 2.6 Sequencing and phylogenetic analysis

Viral genomic RNA was extracted using the MagIso Virus DNA/RNA Isolation Kit (Gbc Biotechnology, Guangzhou, China) and reverse-transcribed using SuperScript^®^ II Reverse Transcriptase (Invitrogen, Carlsbad, CA, USA). The complete genome was amplified from three overlapping PCR fragments, with the 5′ and 3′ termini obtained via rapid amplification of cDNA ends (RACE) using the SMARTer RACE 5′/3′ kit (Takara Bio, Dalian, China). PCR products were sequenced by BioSune Biotechnology Co., Ltd. (Shanghai, China) by Sanger sequencing. All reactions were performed in quintuplicate, and both strands were sequenced for confirmation.

Sequences were analyzed using EditSeq (Lasergene DNASTAR 7.0, DNASTAR Inc., Madison, WI, USA). Homology and phylogenetic relationships were assessed based on complete open reading frame (ORF) and VP1 protein sequences. Alignments were performed using ClustalW, and phylogenetic trees were constructed using the maximum likelihood method in MEGA X with 1, 000 bootstrap replicates (Hall, 2013). Comparisons were made with reference sequences from GenBank to evaluate the genetic diversity and evolutionary relationships.

### 2.7 Animal test

In this study, two independent animal trials were conducted under identical experimental conditions. The first trial (pathogenicity study, N**=**3 per group) aimed to evaluate the pathogenicity of EVG-SH-2024 by accessing clinical signs, virus shedding, tissue viral loads, and histopathology. The second trial (microbiome study) was designed to investigate the effect of EV-G infection on gut microbiota. To ensure adequate statistical power and reliability of the sequencing data, the group size was increased to N=5 mice.

Trial 1: Six EV-G–negative 5-day-old piglets were randomly assigned to two groups (N=3). Piglets in the experimental group received 5 mL of EVG-SH-2024 suspension orally, whereas the controls were administrated with 5 mL of sterile saline. Clinical signs –(appetite, activity, fecal consistency, respiratory symptom)s–) and rectal temperatures were recorded daily. Upon the onset of diarrhea, one piglet from each group was euthanized with pentobarbital overdose and necropsy. Gross gastrointestinal lesions were recorded, and tissues were collected for histopathological evaluation.

Trial 2: Ten EV-G–negative 5-day-old piglets were randomly allocated to two groups (N=5). All other experimental conditions were the same as those in Trial 1. Fecal samples were collected from piglets on day 5 for 16S rRNA sequencing.

### 2.8 Microbial DNA extraction and 16S rRNA sequencing

Total genomic DNA was extracted from fecal samples using the MagBeads FastDNA Kit for feces (MP Biomedicals, CA, USA) according to the manufacturer’s instructions and stored at –20°C. DNA concentration and purity were measured using a NanoDrop NC2000 spectrophotometer (Thermo Fisher Scientific, Waltham, MA, USA), and integrity was confirmed by agarose gel electrophoresis.

The V3–V4 region of the bacterial 16S rRNA gene was amplified using primers 338F (5′-ACTCCTACGGGAGGCAGCA-3′) and 806R (5′-GGACTACHVGGGTWTCTAAT-3′), incorporating sample-specific 7-bp barcodes. PCR reactions (20 µL) contained 5 µL of 5× buffer, 0.25 µL FastPfu DNA Polymerase (5 U/µL), 2 µL dNTPs (2.5 mM), 1 µL of each primer (10 µM), 1 µL DNA template, and 14.75 µL nuclease-free water. Thermal cycling was performed at 98°C for 5 min followed by 25 cycles of 98°C for 30 s, 53°C for 30 s, and 72°C for 45 s; final extension at 72°C for 5min. Amplicons were purified using Vazyme VAHTS™ DNA Clean Beads (Vazyme, Nanjing, China) and quantified using the Quant-iT PicoGreen dsDNA Assay Kit (Invitrogen, Carlsbad, CA, USA). Equimolar amplicons were pooled and sequenced using either Illumina NovaSeq 6000 (paired-end 250 bp, SP Reagent Kit, 500 cycles) or Illumina MiSeq (paired-end 300 bp, Reagent Kit v3) at Shanghai Personal Biotechnology Co., Ltd (Shanghai, China).

Microbiome bioinformatics were performed uusing QIIME2 (Bolyen et al., 2019), following the official tutorials with minor modifications.

### 2.9 Bioinformatics and statistical analysis

Sequence data were analyzed using QIIME2 and R (v4.3.3). Alpha and beta diversity indices were calculated from the ASV table, with Jaccard metrics for beta diversity. Principal component analysis (PCA) was conducted at the genus level. Beta diversity was assessed using UniFrac distances (weighted and unweighted) and visualized by principal coordinate analysis (PCoA). Taxonomic composition and abundance were visualized with MEGAN and GraPhlAn (Asnicar et al., 2015). Venn diagrams were generated using the “VennDiagram” R package to show the shared and unique ASVs. Functional predictions were performed using PICRUSt2 (Phylogenetic investigation of communities by reconstruction of unobserved states) (Schumacher et al., 2025) against the MetaCyc and KEGG databases.

### 2.10 Bioinformatics and statistical analysis

Spearman’s rank-order correlation analysis was conducted using GraphPad 10.3.0 to evaluate the potential associations between the relative abundance of preselected SCFA-producing bacterial taxa and measures of pathogenicity in piglets (Ornstein and Lyhagen, 2016). The pathogenicity indices included clinical diarrhea scores, fecal viral load, and viral load in various tissues. The significance of the correlation coefficients was tested using a two-tailed test, with p < 0.05 deemed statistically significant.

## 3 Results

### 3.1 Identification and cellular biological characterization of an EV-G isolate

**Figure 1:**
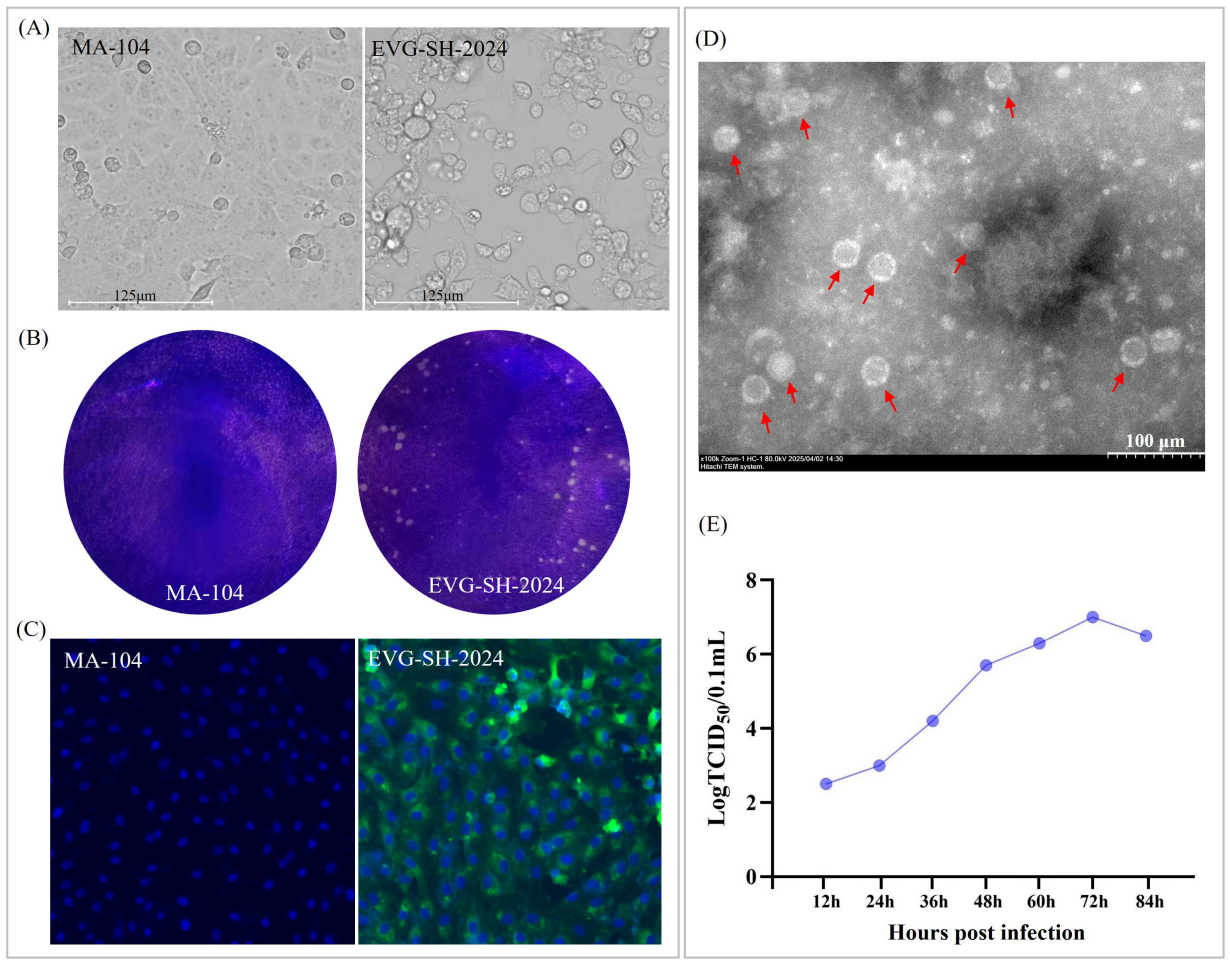
Identification of the growth characteristics of the EVG-SH-2024 strain. CPEs **(A)** and plaque formation **(B)** of EVG-SH-2024 in MA-104 cells were analyzed. Viral infection was further confirmed by indirect immunofluorescesce assay **(C)** and transmission electron microscopic **(D)**. **(E)** Growth kinetics of EVG-SH-2024 in MA-104 cells at an MOI of 0.01.

After four blind passages, an EV-G strain was successfully isolated on MA-104 cells, exhibiting pronounced CPE characterized by rounded, aggregated cells with increased refractility (**Figure 1A**). This isolate, designated EVG-SH-2024, was further purified by plaque assay, yielding uniformly sized white plaques (**Figure 1B**). Immunofluorescence assay confirmed specific green fluorescence within the cytoplasm of MA-104 cells infected with the purified EV-G strain using an EV-G polyclonal antibody (**Figure 1C**). Transmission electron microscopy revealed numerous spherical, non-enveloped, picornavirus-like particles measuring approximately 25–30 nm in diameter (**Figure 1D**). Growth kinetics analysis revealed that EVG-SH-2024 reached a peak titer of 7.6×10^6.50^ 50% tissue culture infectious dose (TCID_50_)/0.1mL at 72 hpi (**Figure 1E**).

### 3.2 Molecular and genetic evolution analysis of EVG-SH-2024

**Figure 2:**
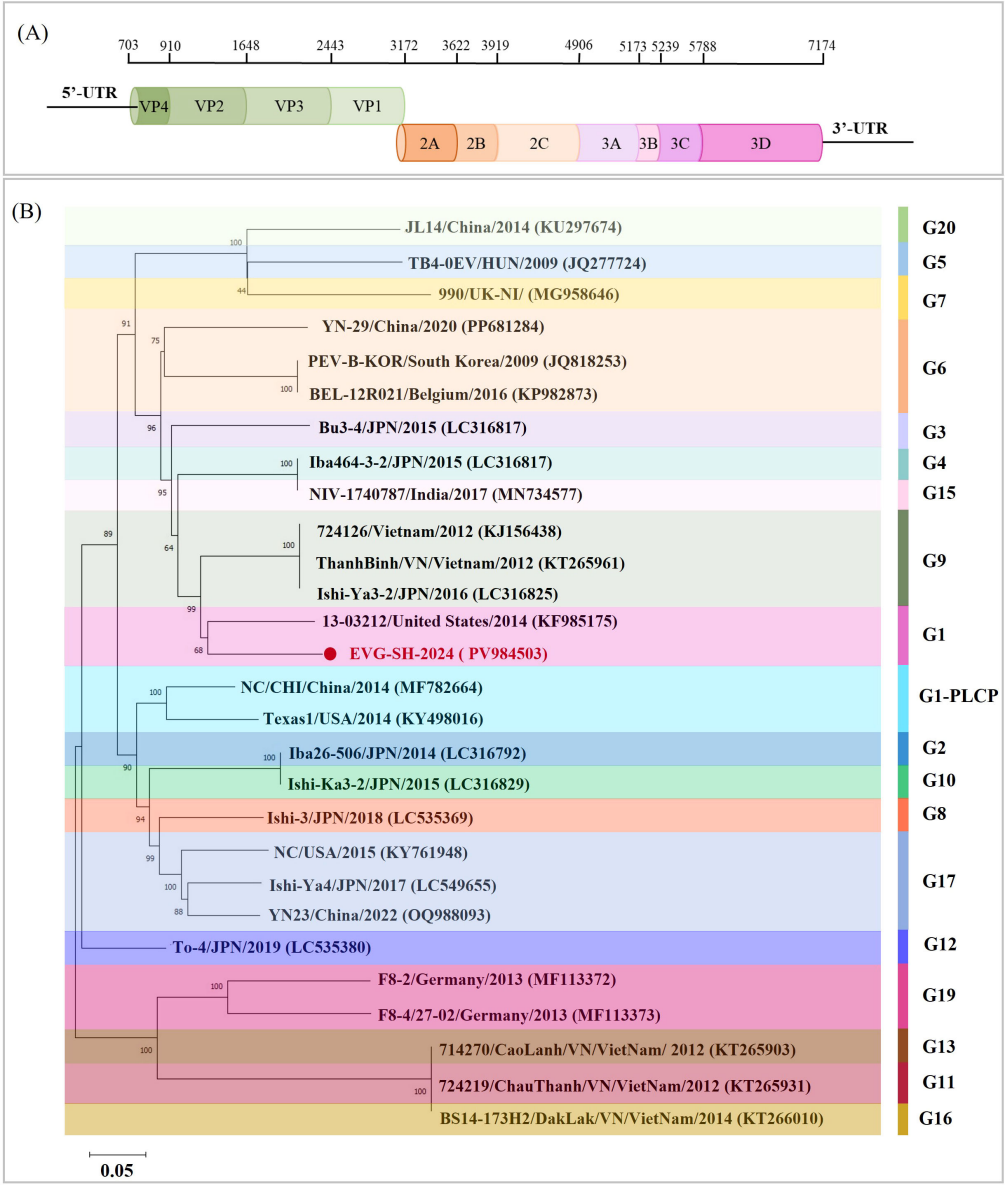
Genomic evolution analysis of EVG-SH-2024 strain. **(A)** Diagram of the genomic structure of EVG-SH-2024. **(B)** Phylogenetic tree based on the whole genomes of the EVG strains. The phylogenetic tree was constructed via the maximum likelihood method in MEGA X with 1, 000 bootstrap replicates. The background colors represent different EV-G genotypes. The EVG-SH-2024 strain is indicated in red font and denoted by a circular dot.

The complete genome sequence of the fifth-passage EVG-SH-2024 strain was determined and deposited in GenBank under the accession number PV984504. The genome was 7, 235 nucleotides (nt) in length, flanked by a 5′-UTR of 702 nt and a 3′-UTR of 60 nt (**Figure 2A**). It comprised a single ORF of 6, 471 nt encoding a 2, 157–amino acid (aa) polyprotein. Phylogenetic analysis of the polyprotein indicated that EVG-SH-2024 clustered most closely with strain 13-03212/United States/2014 (KF985175) and was classified within the G1 genotype (**Figure 2B**). The EV-G1 and EV-G1-PLCP strains segregated into two distinct major clades. Sequence homology comparisons of the polyprotein showed that EVG-SH-2024 shared the highest aa identity (94.7%) with the EV-G1 strain 13-03212/United States/2014, whereas homology with other EV-G strains from GenBank ranged from 78.8% to 94.7%.

**Figure 3:**
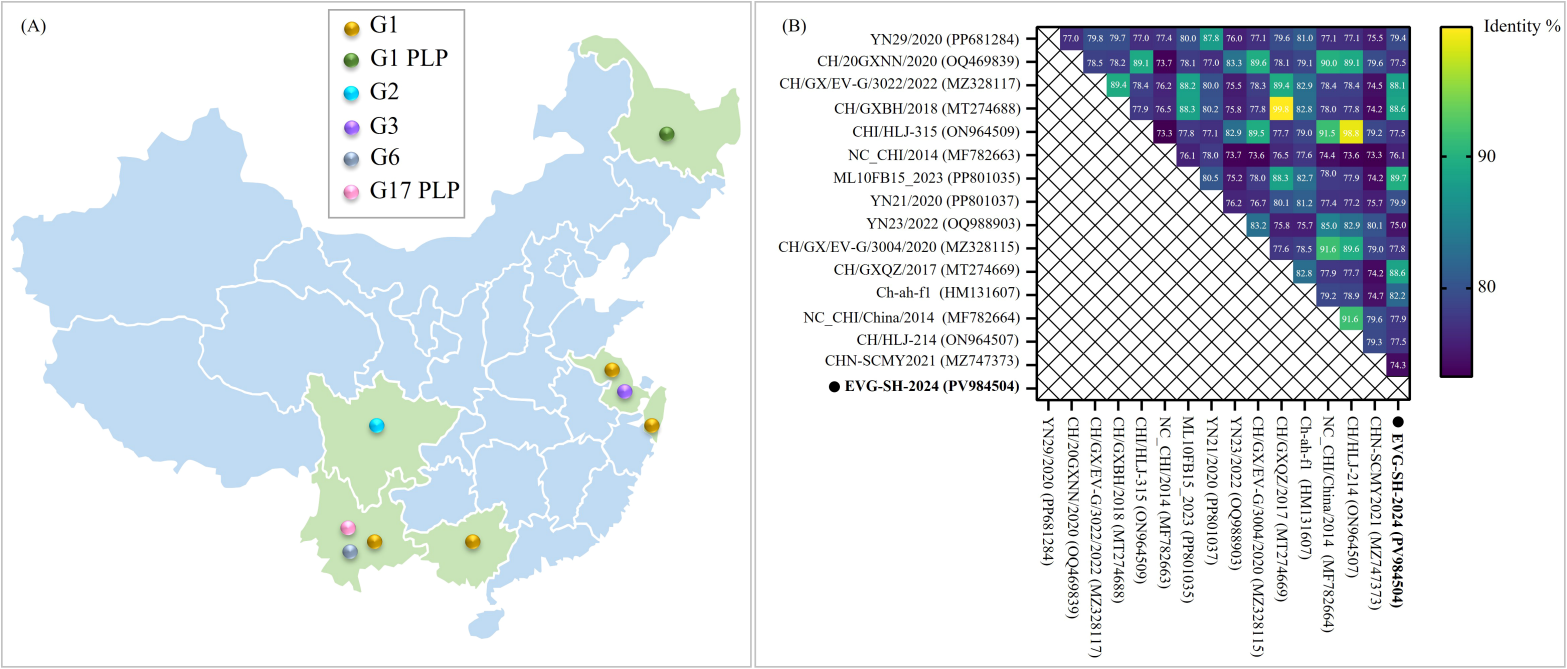
Prevalence and phylogenetic analysis of EV-G strains isolated from China. **(A)** Geographical distribution of distinct EV-G subtypes in China. Distinct subtypes of Chinese EV-G isolates are denoted by circular dots of varying colors. **(B)** Phylogenetic analysis of EVG-SH-2024 and distinct domestic EVG subtypes. Heat map of nucleotide identities between EVG-SH-2024 and 15 reference Chinese strains from GenBank. The nucleotide identity level (top right) was analyzed using MegAlign. The precent identity is represented by the color gradient in the key to the right. The EVG-SH-2024 strain is indicated in black font and denoted by a circular dot.

The identification of EV-G strains in China is limited. Data indicate that the G1 genotype was predominant (**Figure 3A**). This study represents the first report of an EV-G strain in Shanghai, China. In Yunnan, three genotypes (G1, G6, and G17-PLP) have been reported, whereas EV-G G1-PLP, G2, and G3 genotypes have been detected only in Heilongjiang, Sichuan, and Jiangsu, respectively. Comparative genomic analysis revealed low sequence homology among EV-G strains from different provinces in China. EVG-SH-2024 shared nucleotide identities of 82.2%–89.7% with EV-G G1 strains, with the highest nt identity (89.7%) to strain ML10FB15_2023 (PP801035) from Yunnan (**Figure 3B**). Outside the G1 subtype, EVG-SH-2024 showed the highest genomic homology (79.4%–79.9%) with G6 subtype strains but relatively low homology (77.5%–77.9%) with G1-PLP strains (**Figure 3B**).

### 3.3 Recombinant analysis of EVG-SH-2024

**Figure 4:**
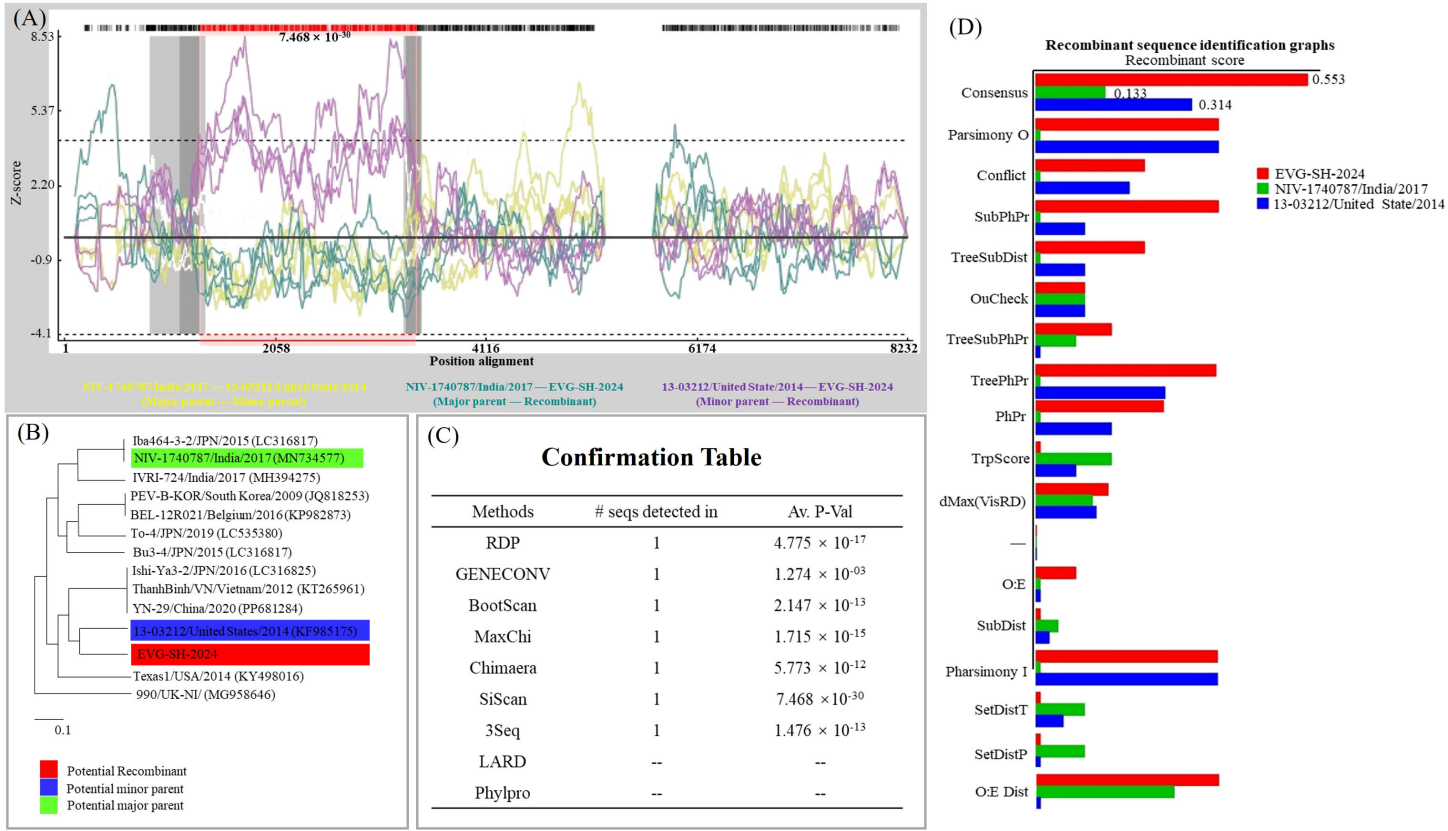
Recombination analyses of the EVG-SH-2024 genome and other EV-G strains. **(A)** The recombination breakpoint analysis of EVG-SH-2024 vs. NIV-1740787/India/2017 (green curve), EVG-SH-2024 vs. 13-03212/United State/2014 (purple curve), 13-03212/United State/2014 vs. NIV-1740787/India/2017 (yellow curve). **(B)** Phylogenetic tree constructed by the neighbor-joining method in MEGAX based on the 1318–3830 region of the EVG-SH-2024 genome. **(C)** Recombination analysis was performed using nine distinct algorithms. Seven approaches confirmed the presence of recombination events in the EVG-SH-2024 genome. **(D)** Scoring of key indicators for the assessment of recombination events. The consensus score of EVG-SH-2024 reached 0.553 > >50%), indicatingd a high level of reliability for the recombination analysis.

Recombination detection program (RDP) analysis of the full-length EVG-SH-2024 genome, together with other EV-G sequences, indicated close relatedness to strain 13-03212/United States/2014 (KF985175) in the genomic region spanning nt 1, 318–3, 830, as determined by SimPlot/SIScan analysis (P ≤ 7.468 × 10^-30^) (**Figure 4A**). Recombination events were strongly supported by seven independent detection methods (P ≤ 1.274 × 10^-3^) (**Figure 4C**). A phylogenetic tree based on the recombinant region (nt 1, 318–3, 830) confirmed that EVG-SH-2024 was most closely related to strain 13-03212/United States/2014 (KF985175) (**Figure 4B**). Recombinant sequence identification via consensus methods yielded identical results (**Figure 4D**). Collectively, SIScan, phylogenetic, and consensus analyses indicated recombination breakpoints at nt positions 1, 318 and 3, 830, with strain NIV-1740787/India/2017 (MN734577) as the major parent and strain 13-03212/United States/2014 (KF985175) as the minor parent, respectively.

**Figure 5:**
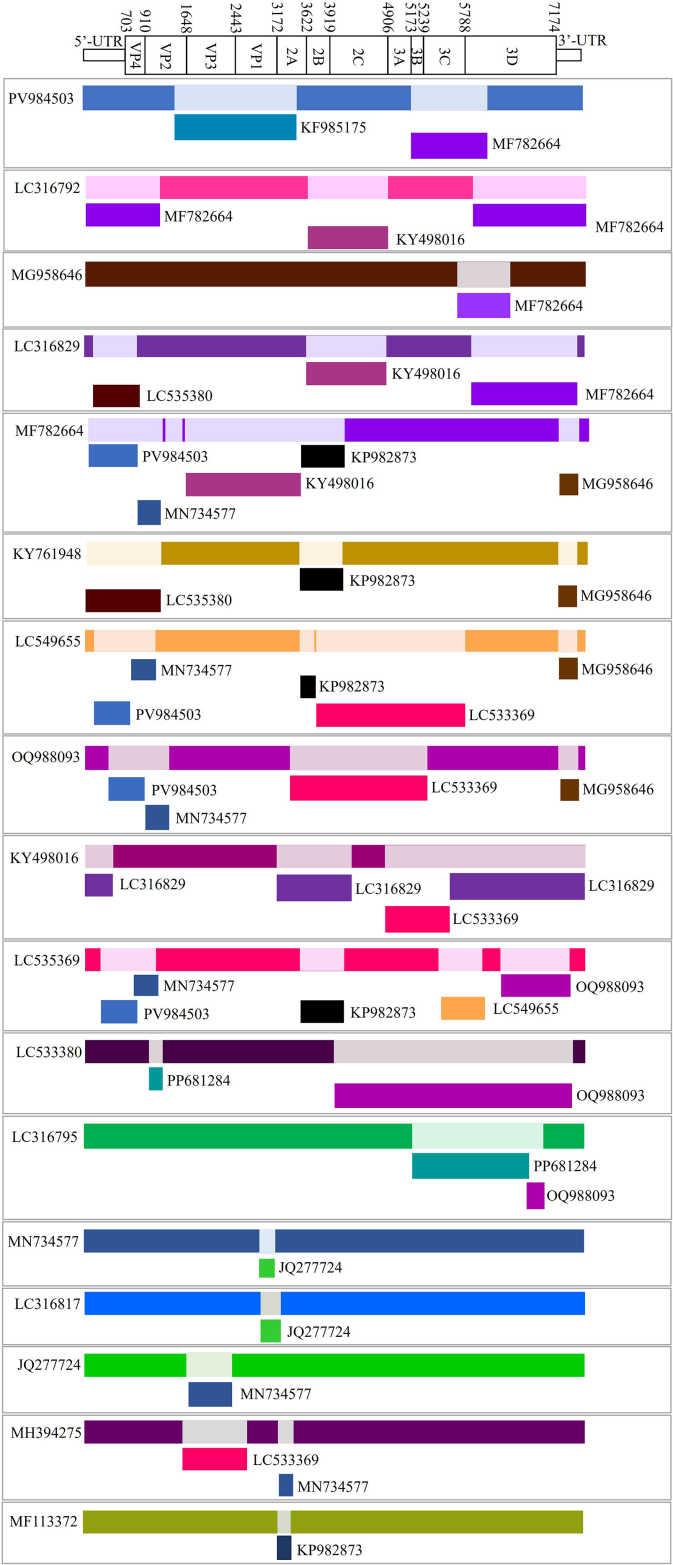
Recombination trend analysis of EV-G Epidemic Strains. Recombination events among the twenty-eight EV-G strains (see **Figure 2**) were analyzed using RDP software. Seventeen high-confidence events, consistently identified by all seven algorithms (RDP, GENECONV, BootScan, MaxChi, Chimaera, SiScan, and 3Seq), were selected for further characterization. Their recombination maps are displayed below, aligned with a reference EVG-SH-2024 (PV984503) genome schematic for comparative analysis.

To explore the characteristics of this recombination event, we performed additional viral recombination analysis on all EV-G strains included in the phylogenetic tree (in **Figure 2**). The results revealed diverse distribution characteristics of recombination breakpoints among different EV-G recombinant strains (**Figure 5**). Notably, the recombination breakpoint in strain EVG-SH-2024 (PV984503) was identified for the first time and has not been detected in other strains. Meanwhile, we observed that several recombination sites recurred in multiple strains. For example, strains LC316792 and LC316829 recombined with KY498016 (in 2A to 2C gene) and MF782664 (in 3D gene) at the same position; strains MF782664, KY761948, LC549655, and LC535369 all recombined with KP982873 (in 2B to 2C gene) in the same region; and strains MF782664, LC549655, O1988093, and LC535369 all recombined with PV984503 and MN734577 in the same region (in 5’-UTR to VP2 gene) (**Figure 5**). It is worth noting that some recombination events involved strains from the same source (e.g., LC316792 and LC316829), suggesting that their genomic recombination may have occurred spontaneously. In contrast, most other recombination events involved strains isolated from different regions. The recombination trend plot reveals a higher propensity for recombination in the non-structural protein region compared to the structural protein region.

### 3.4 Pathogenicity of the EVG-SH-2024 strain on 5-day-old piglets

**Figure 6:**
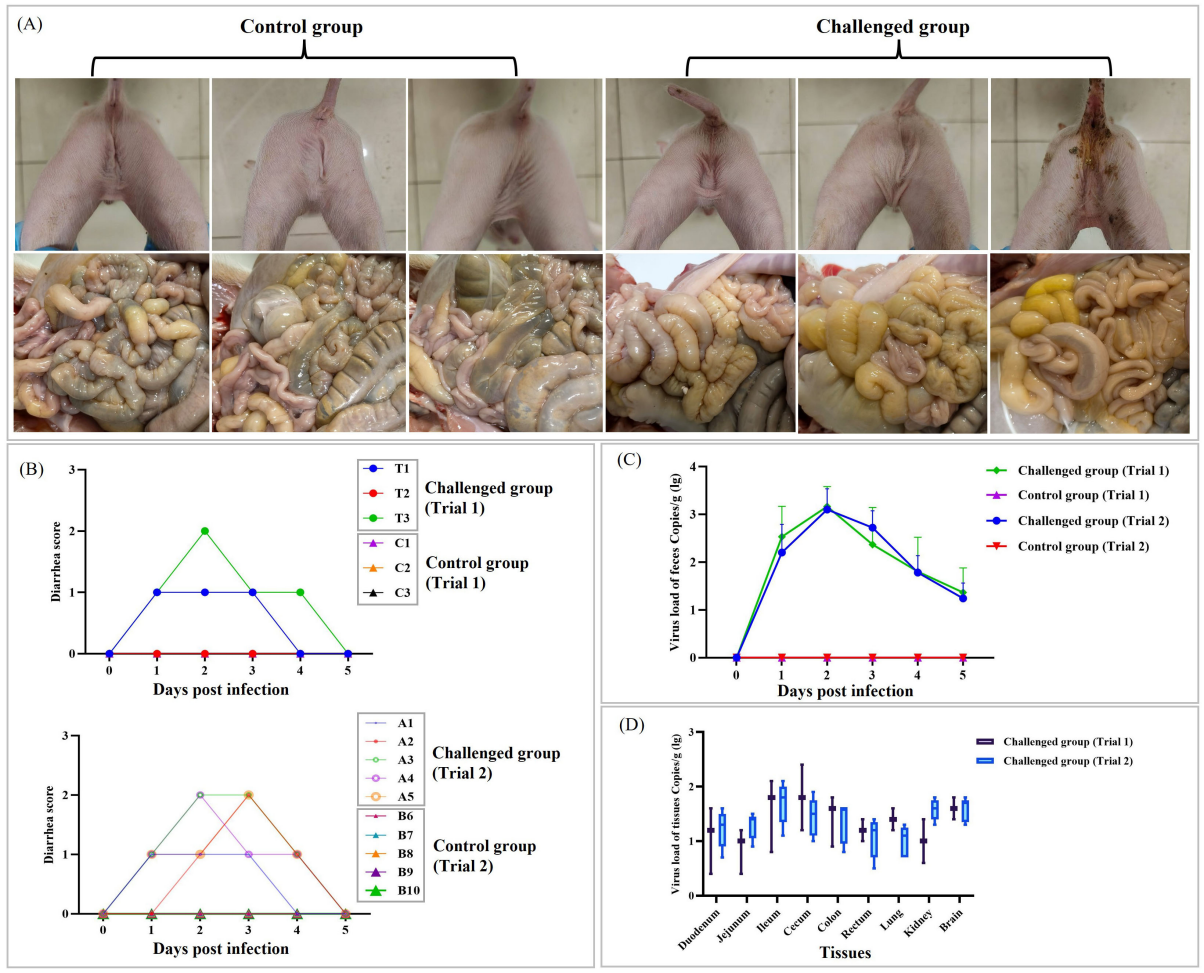
Pathogenicity test of the EVG-SH-2024 strain. **(A)** Clinical symptoms and intestinal tissue changes in piglets in the control and challenged groups were observated. Representative results from 3 piglets in Trial 1 are shown, as clinical and anatomical findings were consistent across both trials. **(B)** Piglet Diarrhea Scoring System. 0 = No diarrhea, 1 = Semi-solid feces, 2 = Porridge-like feces, 3 = Watery diarrhea. Piglet identifiers in the challenged groups were T1–T3 (Trial 1) and A1–A5 (Trial 2). Those in the control groups were C1–C3 (Trial 1) and B6–B10 (Trial 2). **(C)** Fecal virus shedding in the infected piglets from 0 to 5 days post-infection. **(D)** Virus loads in different organs of infected piglets at 5 days post-infection.

To evaluate the pathogenicity of EVG-SH-2024, 5-day-old piglets were orally inoculated with the virus. As in the control group, piglets in challenged group displayed no clinical symptoms throughout both trials (**Figure 6A**). In trial 1, two challenged piglets developed mild diarrhea: one exhibited a fecal score of 1 on days 1–3 dpi, and another had a score of 1 from days 1–4, with a transient increase to 2 on day 2 (**Figure 6B**). In trial 2, the diarrhea scoring pattern was similar: three piglets reached a maximum score of 2 lasting only one day, and one piglet had a maximum score of 2 lasting two days. All five piglets recovered by day 5 post-inoculation (**Figure 6B**).

**Figure 7:**
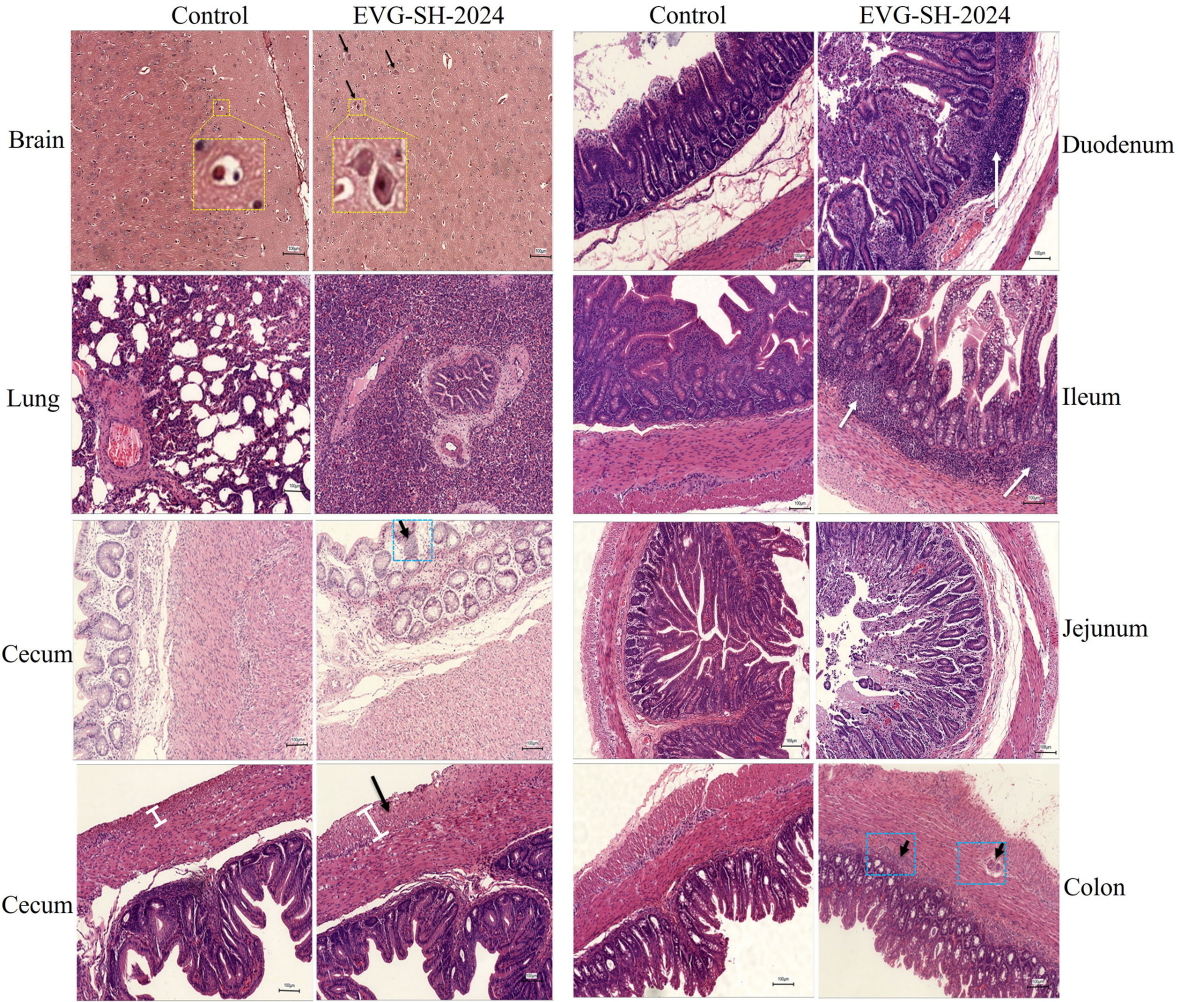
Histopathological analysis of piglets challenged with EVG-SH-2024 and control piglets. Brain, lung, Duodenum, Ileum, Jejunum, Colon, and Cecum tissues were collected at 5 dpi for Hematoxylin and eosin (H&E) staining analysis. The boxes and arrows point to the areas of typical pathological lesions in different tissues.

Viral shedding was monitored daily using RT-PCR with fecal swabs. The shedding trend was consistent across both trials: viral genomic copies in the challenged group increased rapidly within 48 hpi and then gradually declined, with viral loads ranging from 10^1.2^ to 10^3.1^ copies/g (**Figure 6C**). At 5 dpi, all piglets were necropsied, and tissue viral loads were quantified. Consistent with fecal shedding, tissue viral loads showed similar patterns in both trials. EVG-SH-2024 primarily colonized the brain, kidneys, lungs, large intestine, and small intestine (**Figure 6D**). The highest viral loads were detected in the ileum (10^2.1^ copies/g) and cecum (10^2.4^ copies/g), followed by the colon and cerebral parenchyma.Histopathological examination revealed severe multifocal lesions consistent with systemic viral dissemination. Neurological lesions included neuronal vacuolation, cytoplasmic hypertrophy, and nuclear pyknosis with karyorrhexis in the cerebral cortex, indicative of acute neuronal degeneration (**Figure 7**). Pulmonary pathology ranged from disruption of alveolar architecture and pneumocyte hyperplasia to extensive consolidation, with alveolar fusion, interstitial edema, and dense inflammatory infiltration (**Figure 7**). In the gastrointestinal tract, lesions were most severe in the ileum and colon, with dense lymphoplasmacytic infiltration in the submucosa and lymphoid follicle hyperplasia (**Figure 7**). The cecum showed serosal thickening with peritoneal fibrosis, while the colon exhibited transmural inflammation, including myositis, serosal fibrovascular proliferation, and lymphocytic cuffing of the muscularis propria (**Figure 7**).

### 3.5 Shifts in the abundance and composition of gut microbiota due to EVG-SH-2024 infection

**Figure 8:**
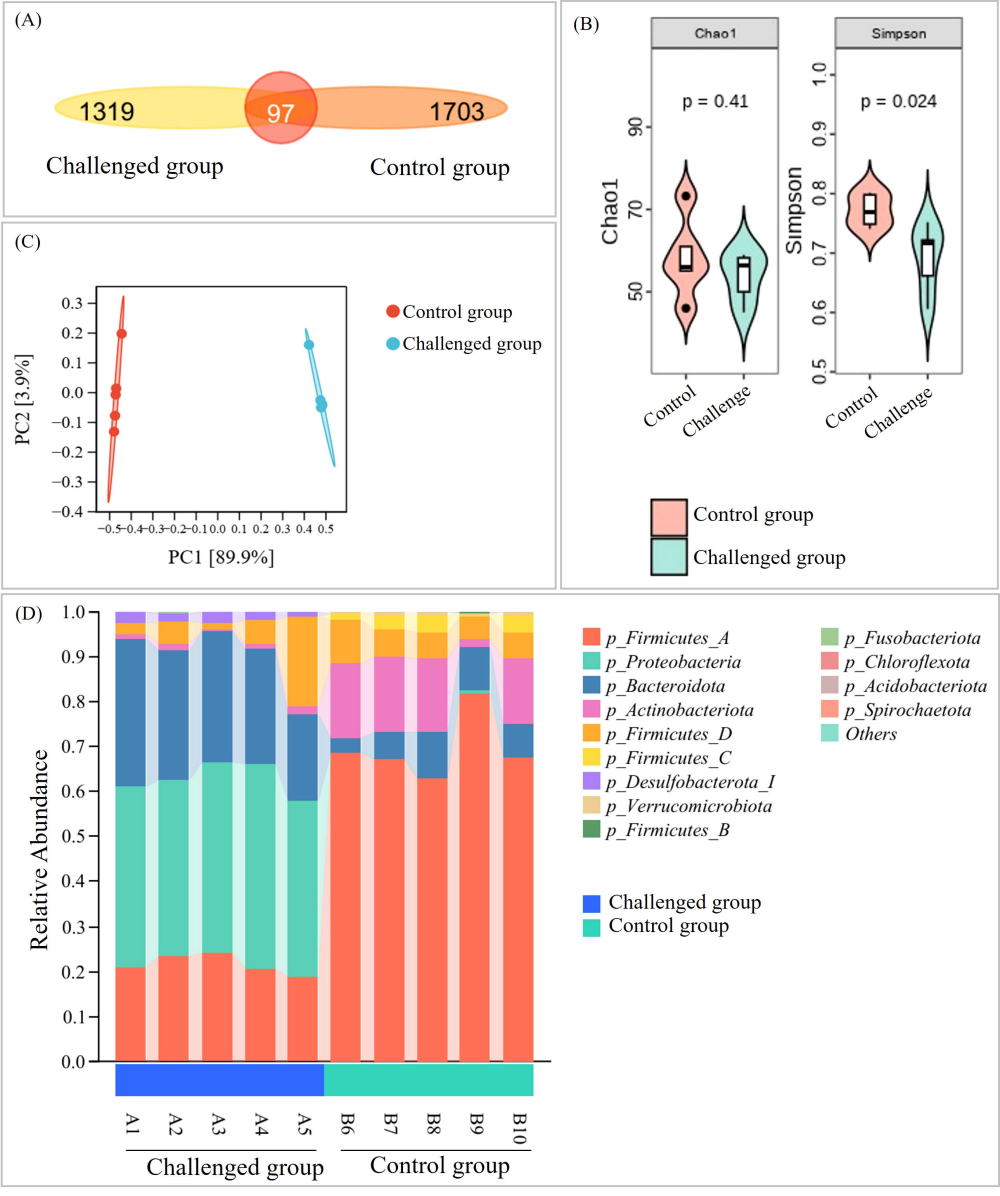
Analysis of the composition and structure of the gut microbiota of piglets in the challenged and control groups. **(A)** Venn diagram of shared OTUs based on sequences with > 97% similarity (N = 5) in each group. The alpha diversity indexes **(B)** and principal coordinate analysis (PCoA) based on Bray_Curtis distances **(C)** of the intestinal microbiota in piglets were compared. **(D)** Bacterial community composition of the two groups using average and Euclidean distance analyses at the phylum level.

Fecal samples from control and challenged piglets at 5 dpi were analyzed by 16S rRNA gene sequencing. A total of 898, 217 high-quality sequences were obtained, with an average length of 415.78 bp. The number of shared and unique OTUs between the control and challenged groups was 1, 703, 1, 319, and 97, respectively (**Figure 8A**). Alpha diversity analysis between the two groups showed no significant difference in richness (Chao index) between the groups; however, the Simpson index revealed a significantly reduced diversity in the challenged group (**Figure 8B**). Beta diversity analysis using Bray–Curtis distances and unweighted UniFrac-based hierarchical clustering demonstrated clear separation of gut bacterial communities between infected and control groups (**Figure 8C**).

EVG-SH-2024 infection induced substantial alterations in the gut microbiota composition, characterized by marked increases in Proteobacteria (42.93%) and Bacteroidota (27.07%) and decreases in Firmicutes_A (21.58%) and Actinobacteriota (1.08%). In the controls, these phyla were present at 0%, 7.30%, 69.59%, and 13.22%, respectively (**Figure 8D**).

### 3.6 Species difference and functional analysis of gut microbiota

**Figure 9:**
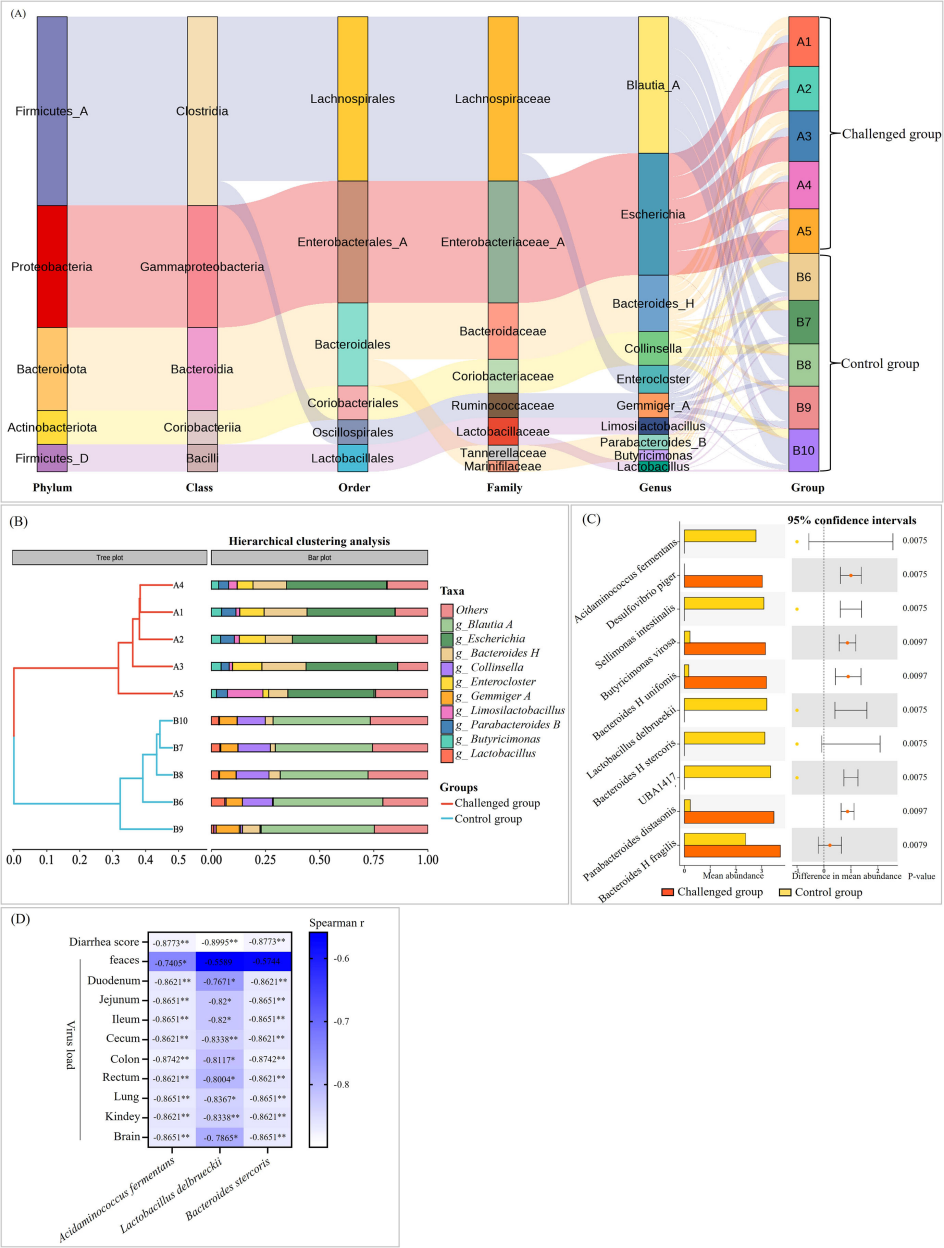
Different species of gut microbiota in the piglets infected with EVG-SH-2024. **(A)** Sankey diagram based on the changes and flow direction of intestinal microbial abundance in piglets infected with EVG-SH-2024. **(B)** Hierarchical clustering analysis at genus level. **(C)** The top 10 differentially abundant species in the two groups, sorted by abundance from highest to lowest. **(D)** Spearman analysis between the abundance of SCFA-producing bacteria and pathogenicity indices (diarrhea score, fecal viral load, and tissue viral load) by GraphPad 10.3.0.

At the family level, EVG-SH-2024 infection significantly reduced the relative abundance of Lachnospiraceae and Ruminococcaceae, while increasing Enterobacteriaceae, Bacteroidaceae, Tannerellaceae, and Marinifilaceae (**Figure 9A**). Genus-level analysis showed that Escherichia, Bacteroides_H, and Enterocoster were enriched in the challenged group, whereas Collinsella, Blautia_A, Gemmiger_A, and Lactobacillus were decreased (**Figure 9B**). These latter genera are important producers of SCFAs that help maintain intestinal microbiota balance. Among the top 10 differentially abundant species, three were significantly downregulated in the challenged group: Acidaminococcus fermentans, Lactobacillus delbrueckii, and Bacteroides_H stercoris—all known SCFA producers (**Figure 9C**). Further correlation analysis between these three downregulated SCFA-producing bacteria and the piglet pathogenicity indices was performed. The results revealed that the relative abundance of Acidaminococcus fermentans exhibited significant negative correlations with the diarrhea score, fecal viral load, and viral loads in all examined tissues. The relative abundances of Lactobacillus delbrueckii and Bacteroides_H stercoris were significantly negatively correlated with the diarrhea score and viral loads across all tissues (**Figure 9D**).

**Figure 10:**
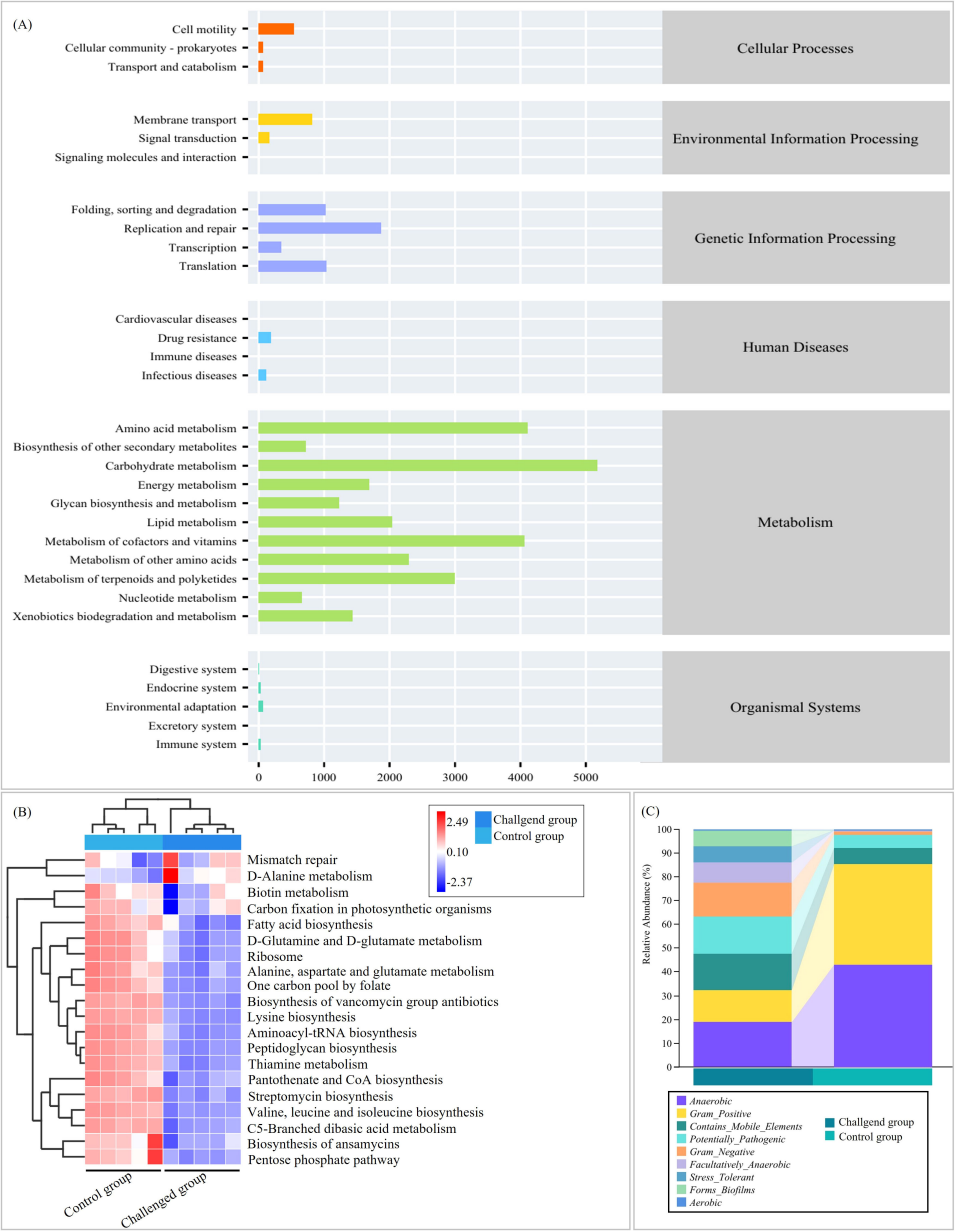
Functional potential prediction of intestinal microbiomes. KEGG pathway **(A)** and COG analysis **(B)** based on PICRUSt2. **(C)** Bacterial phenotype prediction based on bugbase.

Functional predictions using PICRUSt2 (v2.3.0b0) and KEGG enrichment analysis revealed that the majority of annotated pathways were metabolic, followed by genetic information processing (**Figure 10A**). EVG-SH-2024 infection downregulated multiple amino acid metabolism and biosynthesis pathways (**Figure 10B**), potentially compromising intestinal health in piglets. BugBase predictions indicated that the relative abundance of potentially pathogenic bacteria increased from 5.81% in the control group to 15.43% in the challenged group (**Figure 10C**). The abundance of gram-negative bacteria increased substantially (from 1.46% to 14.5%), whereas gram-positive bacteria decreased markedly (from 42.41% to 13.44%). The proportion of facultatively anaerobic bacteria increased, whereas that of strictly anaerobic bacteria declined, further confirming infection-associated dysbiosis (**Figure 10C**).

## 4 Discussion

Similar to other emerging diarrheal pathogens such as astrovirus (AstV), EV-G is detectable in both asymptomatic and diarrheic piglets (Schyns et al., 2025), and frequently co-occurs with other enteric pathogens in clinical settings (Li et al., 2025). This underscores the need for intensified surveillance of viral variants, in-depth pathogenicity studies, and the development of innovative strategies to prevent and control co-infections, thereby reducing the impact of EVG-associated diseases in swine herds.

Most EV-G sequences available in GenBank originate from Japan, the United States, Germany, and other regions, with relatively few from China, likely due to limited clinical surveillance or reporting. Clinically, EV-G-associated diarrhea in piglets is generally mild, and the virus is often found in asymptomatic carriers (Li et al., 2023). This parallels observations for other enteric viruses such as astrovirus, porcine sapelovirus, and porcine teschovirus, which are detected in both healthy and diarrheic pigs. Nonetheless, emerging evidence suggests that these pathogens may act synergistically with PEDV (Wu et al., 2024), justifying further investigation into their epidemiology, genetic diversity, evolutionary dynamics, and pathogenic mechanisms to inform targeted control strategies.

Currently, six EV-G subtypes circulate in China, with EV-G1 being the most prevalent. In this study, full-genome phylogenetic analysis revealed a low nucleotide identity (77.5%–77.9%) between EV-G1 and EV-G1-PLP strains, consistent with previous findings (Knutson et al., 2017). In contrast, EV-G17-PLP showed a higher nucleotide identity (87.5%) with EV-G17 (Knutson et al., 2017). While PLP protein insertions likely contribute to genetic divergence between subtypes, their impact on pathogenicity appears minimal. For example, EV-G17-PLCP (YN-23/2022) caused only mild diarrhea, with viral loads comparable to those in our findings (Li et al., 2023). Similarly, EV-G6 infections primarily presented cskin rashes alongside mild gastrointestinal symptoms (Zhu et al., 2024). In our study, EV-G1 isolates caused gastrointestinal symptoms without dermatological signs, suggesting subtle subtype-specific pathogenic differences that require further investigation. To ensure reproducibility, the pathogenicity experiment was conducted independently twice, first with three piglets per group and then with five. Similar clinical manifestations, characterized by mild diarrhea, were observed in both trials. Furthermore, the viral loads in tissues and organs, as well as the incidence of diarrhea, were consistent across both experiments. This concordance between two independent replicates strongly validates the reliability of our animal model.

The varying clinical pathogenicity of different EV-G strains could be attributed to genetic recombination, which is common among them. This genetic reshuffling represents a plausible explanation for the differences in their virulence and tissue tropism. In this study, we found EVG-SH-2024 exhibited distinct recombination breakpoints between 1, 380–3, 838 nt positions, with closest evolutionary relationships to the U.S. strain 13-03212/2014 and Indian isolates. Since only one EV-G epidemic strain was isolated and identified in this study, the limited data preclude a definitive determination of whether its recombination event was spontaneous or gradual. To preliminarily investigate potential common recombination patterns among EV-G strains, we performed a recombination trend analysis on a set of domestic and international isolates. This analysis revealed that the recombination breakpoint in the EVG-SH-2024 strain was not detected in other strains. However, the recurrence of certain recombination sites in other epidemic strains suggests the existence of potential underlying recombination patterns. The current dataset remains insufficient to robustly confirm or characterize these patterns. Further studies with expanded data are required to more comprehensively and deeply elucidate the recombination and evolutionary dynamics of EV-G. These findings suggest frequent cross-regional genetic exchange and highlight the role of local selective pressure in shaping EV-G diversity. Specifically, EVG-SH-2024 shared recombination breakpoints at aa positions 1, 318 and 3, 830 with strains from the United States and India, indicating a possible U.S. origin. In contrast, the Chinese G17-PLP isolate YN-23/2022 clustered with the Jiangsu G1-PLP strain EVG02/NC CHI/2014 (Li et al., 2023), suggesting a possible derivation of G17-PLP from G1-PLP. Moreover, the Chinese G6 strain YN-29/2022 formed a recombinant clade with the Korean strain PEV-B-KOR (Zhu et al., 2024). Collectively, these results emphasize the high genetic heterogeneity among Chinese EV-G strains, which may be influenced by regional swine trade and cross-border viral transmission.

The intestinal microbiota forms a crucial component of the intestinal barrier and is closely associated with resistance to pathogen invasion (Yang et al., 2020; Shi et al., 2024). To further explore the relationship between EV-G pathogenicity and the gut microbiota, we analyzed bacterial community composition and predicted functional profiles from fecal samples collected before and after infection. In EV-G–uninfected piglets, the gut microbiota exhibited characteristic diversity and stability, shaped predominantly by maternal vertical transmission and environmental exposure. Previous studies have shown that from late gestation through weaning, the intestinal microbiota of sows continuously influences the microbial structure of piglets, conferring distinct maternally derived characteristics (Kiernan et al., 2023). The core gut microbiota of healthy piglets primarily comprises Firmicutes, Bacteroidetes, Proteobacteria, and Actinobacteria, with Firmicutes and Bacteroidetes predominating. These phyla play critical roles in nutrient metabolism, immune modulation, and maintenance of the intestinal mucosal barrier (Zhou et al., 2023). Alpha diversity analysis revealed high richness and evenness in healthy piglets, which is consistent with a mature and stable microbial ecosystem. In contrast, EVG-SH-2024 infection markedly altered microbial composition and diversity, resulting in dysbiosis. Comparative metataxonomic analysis revealed a significant decline in diversity (Simpson index) post-infection, with a pronounced depletion of Firmicutes and Actinobacteriota, and enrichment of Proteobacteria and Bacteroidota. These changes differ from those reported for PEDV infection in piglets. Huang et al. (2018) observed a higher abundance of Firmicutes (35.96%) in PEDV-infected piglets compared with uninfected animals (24.66%), and a lower abundance of Bacteroidetes (8.37%). In co-infections with PEDV and PDCoV, Bacteroidaceae abundance decreased while Actinobacteria increased (Shu et al., 2022). An increase in Proteobacteria is recognized as a microbial signature of intestinal inflammation and dysbiosis (Shin et al., 2015), whereas reductions in Firmicutes and Actinobacteriota are associated with impaired barrier function, weakened immune defenses, and reduced gut resilience (Kothe, 2018; Seong et al., 2018). Firmicutes include many SCFA-producing taxa that supply energy to intestinal epithelial cells (Fusco et al., 2023). In this study, the decline in Firmicutes abundance may partially explain the reduced relative abundance of microbial pathways associated with energy metabolism, secondary metabolite biosynthesis, and amino acid metabolism in the small intestine of infected piglets. At the genus level, Escherichia dominated the challenged group, whereas Blautia_A was predominant in controls. At the species level, Acidaminococcus fermentans, Lactobacillus delbrueckii, and Bacteroides_H stercoris—all SCFA producers—were significantly reduced in infected piglets. Conversely, Desulfovibrio piger, Butyricimonas virosa, Bacteroides_H uniformis, Parabacteroides distasonis, and Bacteroides_H fragilis were significantly increased; these conditional pathogens are associated with virus-induced intestinal inflammation. Further spearman analysis confirmed that the relative abundances of Acidaminococcus fermentans, Lactobacillus delbrueckii, and Bacteroides_H stercoris were significantly negatively correlated with the pathogenicity indicators including diarrhea score, fecal viral load, and tissue viral load. Notably, EVG-SH-2024 infection significantly depleted obligate anaerobes while enriching facultatively anaerobic populations. This microbial imbalance is closely linked to virus-mediated disruption of the intestinal microenvironment, modulation of host immune responses, and impaired intestinal function. The depletion of anaerobes directly reduces SCFA production, thereby weakening intestinal barrier integrity (Fu et al., 2023). As a result, anti-inflammatory capacity declines, and susceptibility to pathogen colonization increases. Meanwhile, certain facultative anaerobes—such as opportunistic Escherichia coli—take advantage of this imbalance to expand their niche. Through toxin production and invasive mechanisms, these bacteria exacerbate intestinal inflammation and sustain diarrheal symptoms, creating a self-reinforcing cycle of dysbiosis and disease progression (Gomez et al., 2022).

The observed microbiome alterations strongly correlate with clinical signs, including diarrhea and vomiting, indicating that gut dysbiosis is a pivotal pathogenic mechanism in EV-G infection. This study advances our understanding of virus–microbiome interactions and provides a scientific basis for microbiota-targeted therapeutic strategies to mitigate EV-G-associated disease. However, this study has several limitations. First, the sample size for gut microbiota analysis was relatively small, and the study lacked comparison with clinical samples. In future work, we will focus on clinical EV-G infection cases to further validate the relationship between our laboratory findings and clinical data. Second, our analysis of the gut microbiota remained preliminary. While we observed a reduction in the abundance of short-chain fatty acid (SCFA)-producing bacteria, we did not perform targeted metabolomics to directly confirm the decrease in SCFA levels. Nevertheless, based on previous studies of other enteroviruses, it is reasonable to hypothesize that a decline in SCFA-producing bacteria may lead to reduced SCFA production. In subsequent studies, we will identify which specific SCFAs are most significantly affected and investigate how SCFAs influence EV-G replication, thereby further elucidating the mechanism by which EV-G infection contributes to gut microbiota dysbiosis.

Overall, our findings have both theoretical and practical implications: they define the molecular and phylogenetic characteristics of Shanghai-dominant EV-G strains and elucidate the microecological mechanisms underlying EV-G pathogenesis through the lens of gut dysbiosis. These results present a compelling rationale for developing microbiota-modulating interventions to control EV-G-induced disease in swine populations.

## Data Availability

The data presented in this study are deposited in the NCBI repository, accession number PRJNA1314931.

## References

[B1] Asnicar F. Weingart G. Tickle T. L. Huttenhower C. Segata N. (2015). Compact graphical representation of phylogenetic data and metadata with GraPhlAn. Peer. J. 3, e1029. doi: 10.7717/peerj.1029, PMID: 26157614 PMC4476132

[B2] Bhat S. Ansari M. I. Kattoor J. J. Sircar S. Dar P. S. Deol P. . (2024). Emerging porcine Enterovirus G infections, epidemiological, complete genome sequencing, evolutionary and risk factor analysis in India. Virology 590, 109906. doi: 10.1016/j.virol.2023.109906, PMID: 38096748

[B3] Bolyen E. Rideout J. R. Dillon M. R. Bokulich N. A. Abnet C. C. Al-Ghalith G. A. . (2019). Author Correction: Reproducible, interactive, scalable and extensible microbiome data science using QIIME 2. Nat. Biotechnol. 37, 1091. doi: 10.1038/s41587-019-0252-6, PMID: 31399723

[B4] Chen Y. M. Yeh C. A. Lin W. H. Lin C. N. Chiou M. T. (2025). Dynamic alterations of the intestinal microbiome and metabolome during transmissible gastroenteritis virus infection in weaned pigs. Microb. Pathog. 206, 107705. doi: 10.1016/j.micpath.2025.107705, PMID: 40383241

[B5] Fu Y. Lyu J. Wang S. (2023). The role of intestinal microbes on intestinal barrier function and host immunity from a metabolite perspective. Front. Immunol. 14, 1277102. doi: 10.3389/fimmu.2023.1277102, PMID: 37876938 PMC10591221

[B6] Fusco W. Lorenzo M. B. Cintoni M. Porcari S. Rinninella E. Kaitsas F. . (2023). Short-chain fatty-acid-producing bacteria: key components of the human gut microbiota. Nutrients 15, 2211. doi: 10.3390/nu15092211, PMID: 37432351 PMC10180739

[B7] Gomez D. E. Li L. Goetz H. Macnicol J. Gamsjaeger L. Renaud D. L. (2022). Calf diarrhea is associated with a shift from obligated to facultative anaerobes and expansion of lactate-producing bacteria. Front. Vet. Sci. 9, 846383. doi: 10.3389/fvets.2022.846383, PMID: 35392114 PMC8981386

[B8] Hall B. G. (2013). Building phylogenetic trees from molecular data with MEGA. Mol. Biol. Evol. 30, 1229–1235. doi: 10.1093/molbev/mst012, PMID: 23486614

[B9] Huang M. Z. Wang S. Y. Wang H. Cui D. A. Yang Y. J. Liu X. W. . (2018). Differences in the intestinal microbiota between uninfected piglets and piglets infected with porcine epidemic diarrhea virus. PLoS. One 13, e0192992. doi: 10.1371/journal.pone.0192992, PMID: 29447243 PMC5814011

[B10] Ibrahim Y. M. Zhang W. Wang X. Werid G. M. Fu L. Yu H. . (2023). Molecular characterization and pathogenicity evaluation of enterovirus G isolated from diarrheic piglets. Microbiol. Spectr. 11, e0264323. doi: 10.1128/spectrum.02643-23, PMID: 37830808 PMC10715025

[B11] Ibrahim Y. M. Zhang W. Werid G. M. Zhang H. Pan Y. Zhang L. . (2022). Characterization of parainfluenza virus 5 from diarrheic piglet highlights its zoonotic potential. Transbound Emerg. Dis. 69, e1510–e1525. doi: 10.1111/tbed.14482, PMID: 35179303

[B12] Ishizaka A. Koga M. Mizutani T. Yamayoshi S. Iwatsuki-Horimoto K. Adachi E. . (2024). Association of gut microbiota with the pathogenesis of SARS-CoV-2 Infection in people living with HIV. BMC. Microbiol. 24, 6. doi: 10.1186/s12866-023-03157-5, PMID: 38172680 PMC10763188

[B13] Kiernan D. P. O’doherty J. V. Sweeney T. (2023). The effect of maternal probiotic or synbiotic supplementation on sow and offspring gastrointestinal microbiota, health, and performance. Anim. (Basel). 13, 2996. doi: 10.3390/ani13192996, PMID: 37835602 PMC10571980

[B14] Knowles N. J. Buckley L. S. Pereira H. G. (1979). Classification of porcine enteroviruses by antigenic analysis and cytopathic effects in tissue culture: description of 3 new serotypes. Arch. Virol. 62, 201–208. doi: 10.1007/BF01317552, PMID: 229804

[B15] Knutson T. P. Velayudhan B. T. Marthaler D. G. (2017). A porcine enterovirus G associated with enteric disease contains a novel papain-like cysteine protease. J. Gen. Virol. 98, 1305–1310. doi: 10.1099/jgv.0.000799, PMID: 28590234 PMC5656790

[B16] Kothe E. (2018). Special focus: actinobacteria. J. Basic. Microbiol. 58, 719. doi: 10.1002/jobm.201870028, PMID: 30175507

[B17] Krumbholz A. Dauber M. Henke A. Birch-Hirschfeld E. Knowles N. J. Stelzner A. . (2002). Sequencing of porcine enterovirus groups II and III reveals unique features of both virus groups. J. Virol. 76, 5813–5821. doi: 10.1128/JVI.76.11.5813-5821.2002, PMID: 11992011 PMC137026

[B18] Li Z. H. Li Z. R. Zhu P. Zhang Z. X. Song J. L. (2023). First identification and pathogenicity evaluation of an EV-G17 strain carrying a torovirus papain-like cysteine protease (PLCP) gene in China. Viruses 15, 1747. doi: 10.3390/v15081747, PMID: 37632087 PMC10459844

[B19] Li B. Shi K. Shi Y. Feng S. Yin Y. Lu W. . (2025). A quadruplex RT-qPCR for the detection of porcine sapelovirus, porcine kobuvirus, porcine teschovirus, and porcine enterovirus G. Anim. (Basel). 15, 1008. doi: 10.3390/ani15071008, PMID: 40218401 PMC11987865

[B20] Mi X. Yang C. Lu Y. Wang H. Qin Q. Chen R. . (2021). Isolation, identification, and evaluation of the pathogenicity of a porcine enterovirus G isolated from China. Front. Vet. Sci. 8, 712679. doi: 10.3389/fvets.2021.712679, PMID: 34368288 PMC8339413

[B21] Ng H. Y. Leung W. K. Cheung K. S. (2023). Association between gut microbiota and SARS-CoV-2 infection and vaccine immunogenicity. Microorganisms 11, 452. doi: 10.3390/microorganisms11020452, PMID: 36838417 PMC9961186

[B22] Ornstein P. Lyhagen J. (2016). Asymptotic properties of spearman’s rank correlation for variables with finite support. PLoS. One 11, e0145595. doi: 10.1371/journal.pone.0145595, PMID: 26730491 PMC4701424

[B23] Paul J. K. Azmal M. Haque A. Meem M. Talukder O. F. Ghosh A. (2025). Unlocking the secrets of the human gut microbiota: Comprehensive review on its role in different diseases. World. J. Gastroenterol. 31, 99913. doi: 10.3748/wjg.v31.i5.99913, PMID: 39926224 PMC11718612

[B24] Schumacher S. M. Doyle W. J. Hill K. Ochoa-Reparaz J. (2025). PICRUSt2 analysis of fecal microbiome associated with a murine model of multiple sclerosis. FASEB. Bioadv. 7, e70029. doi: 10.1096/fba.2025-00060, PMID: 40654331 PMC12246385

[B25] Schyns M. Van Den Braak R. Peijnenborg J. Coppens S. Deijs M. Burggraaff M. . (2025). Characterization of the enteric virome of clinically healthy pigs around weaning on commercial farms in the Netherlands using next generation sequencing and qPCR. Porcine. Health Manage. 11, 41. doi: 10.1186/s40813-025-00446-5, PMID: 40708015 PMC12291374

[B26] Seong C. N. Kang J. W. Lee J. H. Seo S. Y. Woo J. J. Park C. . (2018). Taxonomic hierarchy of the phylum Firmicutes and novel Firmicutes species originated from various environments in Korea. J. Microbiol. 56, 1–10. doi: 10.1007/s12275-018-7318-x, PMID: 29299839

[B27] Shi Y. Li B. Cheng J. Tao J. Tang P. Jiao J. . (2024). Microbial community and metabolome analysis of the porcine intestinal damage model induced by the IPEC-J2 cell culture-adapted porcine deltacoronavirus (PDCoV) infection. Microorganisms 12, 874. doi: 10.3390/microorganisms12050874, PMID: 38792704 PMC11124095

[B28] Shin N. R. Whon T. W. Bae J. W. (2015). Proteobacteria: microbial signature of dysbiosis in gut microbiota. Trends. Biotechnol. 33, 496–503. doi: 10.1016/j.tibtech.2015.06.011, PMID: 26210164

[B29] Shu X. Han F. Hu Y. Hao C. Li Z. Wei Z. . (2022). Co-infection of porcine deltacoronavirus and porcine epidemic diarrhoea virus alters gut microbiota diversity and composition in the colon of piglets. Virus. Res. 322, 198954. doi: 10.1016/j.virusres.2022.198954, PMID: 36198372

[B30] Song D. Peng Q. Chen Y. Zhou X. Zhang F. Li A. . (2017). Altered gut microbiota profiles in sows and neonatal piglets associated with porcine epidemic diarrhea virus infection. Sci. Rep. 7, 17439. doi: 10.1038/s41598-017-17830-z, PMID: 29234140 PMC5727058

[B31] Walker P. J. Siddell S. G. Lefkowitz E. J. Mushegian A. R. Adriaenssens E. M. Alfenas-Zerbini P. . (2021). Changes to virus taxonomy and to the International Code of Virus Classification and Nomenclature ratified by the International Committee on Taxonomy of Viruses, (2021). Arch. Virol. 166, 2633–2648. doi: 10.1007/s00705-021-05156-1, PMID: 34231026

[B32] Wang Y. Zhang W. Liu Z. Fu X. Yuan J. Zhao J. . (2018). Full-length and defective enterovirus G genomes with distinct torovirus protease insertions are highly prevalent on a Chinese pig farm. Arch. Virol. 163, 2471–2476. doi: 10.1007/s00705-018-3875-x, PMID: 29786119

[B33] Wu S. Gou F. Meng J. Jin X. Liu W. Ding W. . (2024). Porcine kobuvirus enhances porcine epidemic diarrhea virus pathogenicity and alters the number of intestinal lymphocytes in piglets. Vet. Microbiol. 293, 110100. doi: 10.1016/j.vetmic.2024.110100, PMID: 38718527

[B34] Xiao D. Zhang L. Li S. Liang Y. Wu R. Wen Y. . (2023). Characterization, phylogenetic analysis, and pathogenicity of a novel genotype 2 porcine Enterovirus G. Virus. Res. 335, 199185. doi: 10.1016/j.virusres.2023.199185, PMID: 37532142 PMC10448215

[B35] Yang S. Li Y. Wang B. Yang N. Huang X. Chen Q. . (2020). Acute porcine epidemic diarrhea virus infection reshapes the intestinal microbiota. Virology 548, 200–212. doi: 10.1016/j.virol.2020.07.001, PMID: 32763491 PMC7353907

[B36] Zhang Y. Si L. Gao J. Shu X. Qiu C. Zhang Y. . (2024). Serial passage of PDCoV in cell culture reduces its pathogenicity and its damage of gut microbiota homeostasis in piglets. mSystems 9, e0134623. doi: 10.1128/msystems.01346-23, PMID: 38349151 PMC10949489

[B37] Zhou H. Yu B. Sun J. Chen H. Liu Z. Ge L. . (2023). Gut microbiota absence and transplantation affect diarrhea: an investigation in the germ-free piglet model. Anim. Biotechnol. 34, 3971–3977. doi: 10.1080/10495398.2023.2248200, PMID: 37906091 PMC13353480

[B38] Zhu P. Li Z. H. Li Z. R. Zhang Z. X. Song J. L. (2024). First isolation, identification, and pathogenicity evaluation of an EV-G6 strain in China. Front. Vet. Sci. 11, 1431180. doi: 10.3389/fvets.2024.1431180, PMID: 39113722 PMC11304196

[B39] Zhu L. T. Zhao L. Zhu Y. Xu X. L. Lin J. J. Duan Y. F. . (2025). Disruption and adaptation: infant gut microbiota’s dynamic response to SARS-CoV-2 infection. Microbiome 13, 72. doi: 10.1186/s40168-025-02029-6, PMID: 40069800 PMC11895207

